# Supplementary figures and images for: Exposing Hidden Alternative Backbone Conformations in X-ray Crystallography Using qFit
Source: PLoS Comput Biol. 2015 Oct 27;11(10):e1004507. doi: 10.1371/journal.pcbi.1004507 (PMC4624436; doi:10.1371/journal.pcbi.1004507)

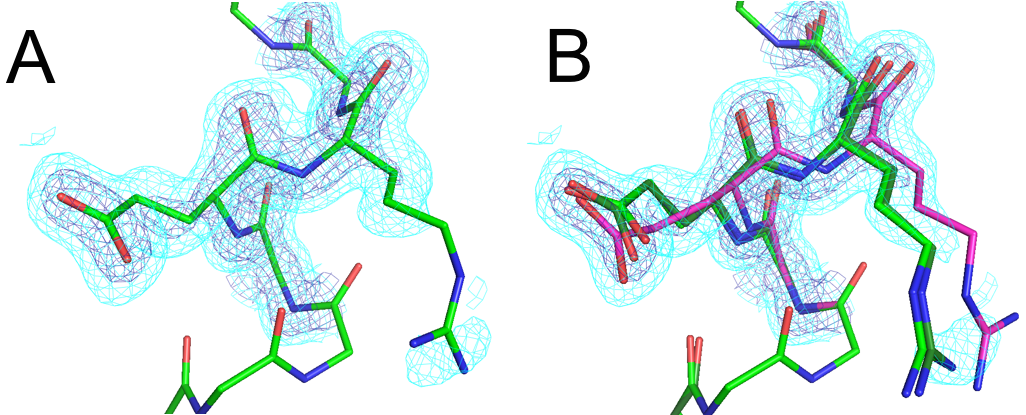

Supplement: S1 Fig — (A) Residues 142–145 in CypA are modeled with a single conformation in the single-conformer structure (PDB ID 3k0n). The model is a reasonable fit to the 2mFo-DFc electron density contoured at 1.0 σ (cyan) and 2.5 σ (dark blue), which is slightly anisotropic for the central carbonyl oxygen. (B) The multiconformer qFit model, on the other hand, includes three alternative conformations with backbones related by a shear-like motion to explain the electron density. Each shear end-state (greens vs. purple) is allocated about 50% occupancy. The multiconformer model adds a second rotamer (purple) in addition to the original rotamer (greens) for Glu143 (left-hand-side of panel) and sweeps the Arg144 sidechain sideways (right-hand-side of panel). (TIFF) [file pcbi.1004507.s001.tiff]

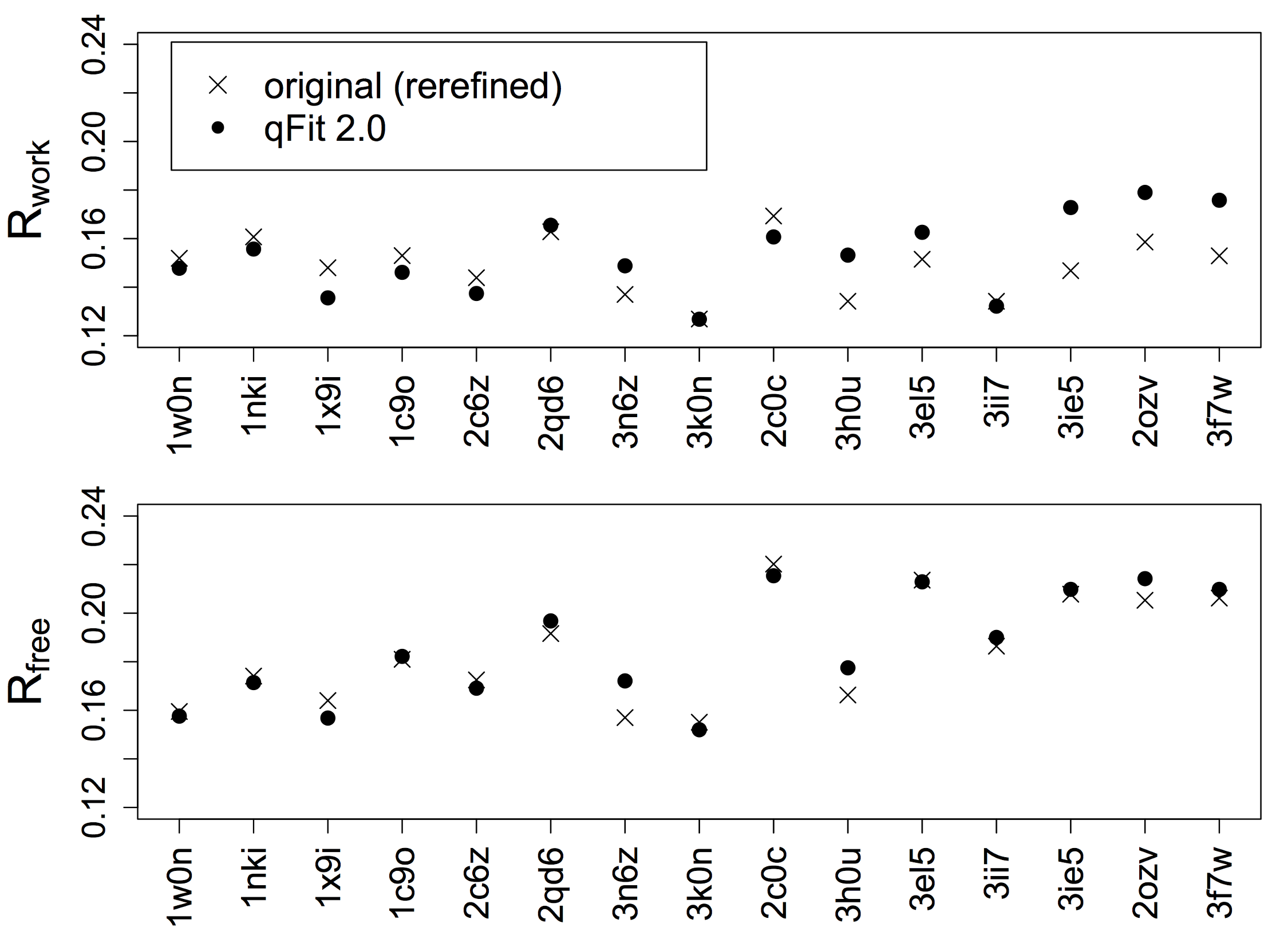

Supplement: S2 Fig — Rwork and Rfree are plotted vs. PDB ID sorted from high to low resolution. X’s indicate original structures rerefined without automated addition and removal of water molecules, and filled circles indicate qFit 2.0 models. (TIFF) [file pcbi.1004507.s002.tiff]
